# Colorectal neuroendocrine tumors: A case series

**DOI:** 10.1016/j.ijscr.2020.06.030

**Published:** 2020-06-12

**Authors:** Warsinggih, Liliyanto, Prihantono, Gusti Deasy Wilda Ariani, Muhammad Faruk

**Affiliations:** a Division of Digestive, Department of Surgery, Faculty of Medicine, Hasanuddin University Makassar, Indonesia; b Division of Oncology, Department of Surgery, Faculty of Medicine, Hasanuddin University Makassar, Indonesia; c Department of Anatomic Pathology, Faculty of Medicine, Hasanuddin University Makassar, Indonesia; d Department of Surgery, Faculty of Medicine, Hasanuddin University Makassar, Indonesia

**Keywords:** Neuroendocrine tumor, Colorectal cancer, Peritonitis, Large bowel obstruction, Case series

## Abstract

**Introduction:**

Neuroendocrine tumors (NET) of the colon and sigmoid colon are uncommon compared to colorectal adenocarcinoma. Few reports have been made of NET of the colon and sigmoid colon that presents with peritonitis and large bowel obstruction.

**Case presentation:**

Here, we report two cases of NET of the colon and sigmoid colon, which were diagnosed and treated at our institution. In our first case, a 66-year-old man with a history of abdominal distension was diagnosed with NET via histopathology of the sigmoid colon. The second case involved a 45-year-old woman with the chief complaints of abdominal distention and inability to defecate; specimen histopathology of the descending colon showed neuroendocrine carcinoma features. Clinical outcome was very poor in our patients: eight months after the resection, the second patient demonstrated a sign of metastasis on the liver.

**Conclusion:**

An uncommon case of colon and sigmoid colon carcinoma with neuroendocrine and diagnostic difficulties precludes an exact description of the initial diagnostic criteria and management. Thus, our case series offers an overview of initial symptoms, radiological and histopathological features for early diagnosis, and proper management of NET.

## Highlights

• Neuroendocrine tumors (NET) of the colon and sigmoid colon are uncommon compared to colorectal adenocarcinoma.
• The incidence of NETs has increased, probably due to improved diagnosis and the availability of very specific and sensitive modalities.
• Here, we report a serial case of Gastrointestinal NET (GI-NET) found in the descending colon and sigmoid colon.

## 1 Introduction

Neuroendocrine carcinoma (NEC) of the large intestine and rectum is an uncommon type of neuroendocrine tumor (NET) that accounts for <1% of all colorectal cancer [[1], [2], [3]]. The clinical development of NEC includes very destructive and aggressive growth, followed by rapid spread; it is accompanied by a significant tendency to metastasis [2]. These aggressive tumors cause serious health problems, such as colonic obstruction and exclusion of internal organs [4]; clinically, it results in a poor prognosis [2].

The incidence of NETs has increased, probably due to improved diagnosis and the availability of very specific and sensitive modalities, like endoscopy, computed tomography (CT scan), magnetic resonance imaging (MRI), ultrasonography (USG), and scintigraphy [5].

The incidence rate by age increased sixfold from 1973 (1.09 per 100,000) to 2012 (6.98 per 100,000) [6]. In Indonesia, accurate data on this tumor have not been obtained. Here, we report a serial case of Gastrointestinal NET (GI-NET) found in the descending colon and sigmoid in a patient in our referral hospital. This case series has been reported in line with the PROCESS criteria [7].

## 2 Case presentation

### 2.1 Case 1

**Fig. 1:**
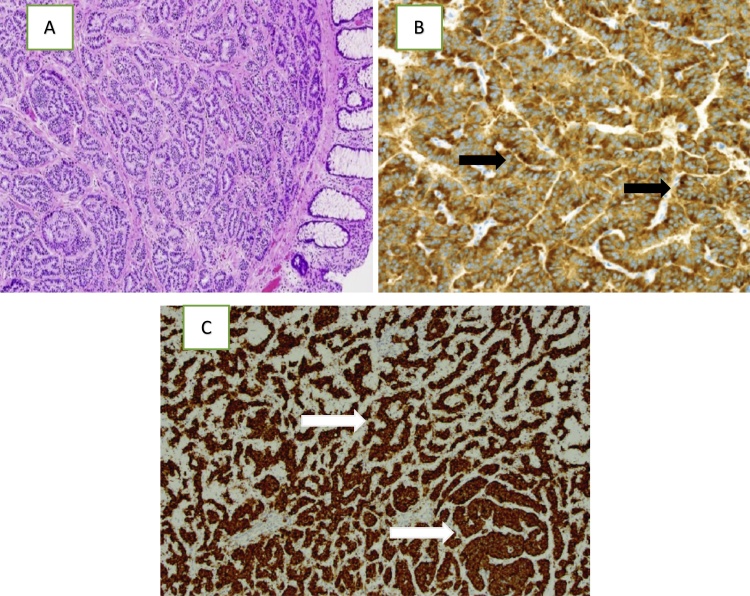
Histopathology slide showing: A) NET invading the submucosa (Hematoxylin-eosin staining, 4x); B) positive result for chromogranin A in neoplastic cells (the nuclei are blue and cytoplasm was brown) (black arrows); C) positive brown-stained cytoplasm for synaptophysin in neoplastic cells (white arrows).

A 66-year-old man was admitted to our hospital after a previous laparotomy surgery in a tertiary hospital. Before the surgery, the patient had abdominal distension for three days and diarrhea for two weeks, which led to his admission to the hospital. The patient was diagnosed with peritonitis in the tertiary hospital. On August 13th, 2018, sigmoidectomy (Hartmann’s procedure) and primary closure of perforated caecum was performed in the tertiary hospital. The post-surgical diagnosis was peritonitis caused by perforated caecum and distal sigmoid colon tumors. The histopathology results on August 29th, 2018, showed neuroendocrine carcinoma with a near incision edge; all lymph node specimens were tumor-free (Fig. 1).

The patient came to our institution on November 12th, 2018, and reevaluation was conducted; abdominal ultrasound showed no sign of metastasis as well as other intra-abdominal organs within normal limits. Thoracic x-ray examination showed no sign of metastasis. The lopography result was within normal limits: the distance of the rectosigmoid colon from the distal descending colon was aproximately 9.5 cm. Laboratory tests showed hemoglobin 12.3 g/dl; white blood cell 16.0 × 10^3^/ul; platelets 493,000/ul; albumin, liver function test values, and other biochemical parameters within normal limits; and carcinoembryonic antigen (CEA) tumor markers within normal levels. We operated close the stoma. The post-operative period was uneventful, with removal of a drain on postoperative day 4, and the patient was discharged on the 5th post-operative day. Patient showed no signs of recurrence during the two-year follow-up period.

### 2.2 Case 2

**Fig. 2:**
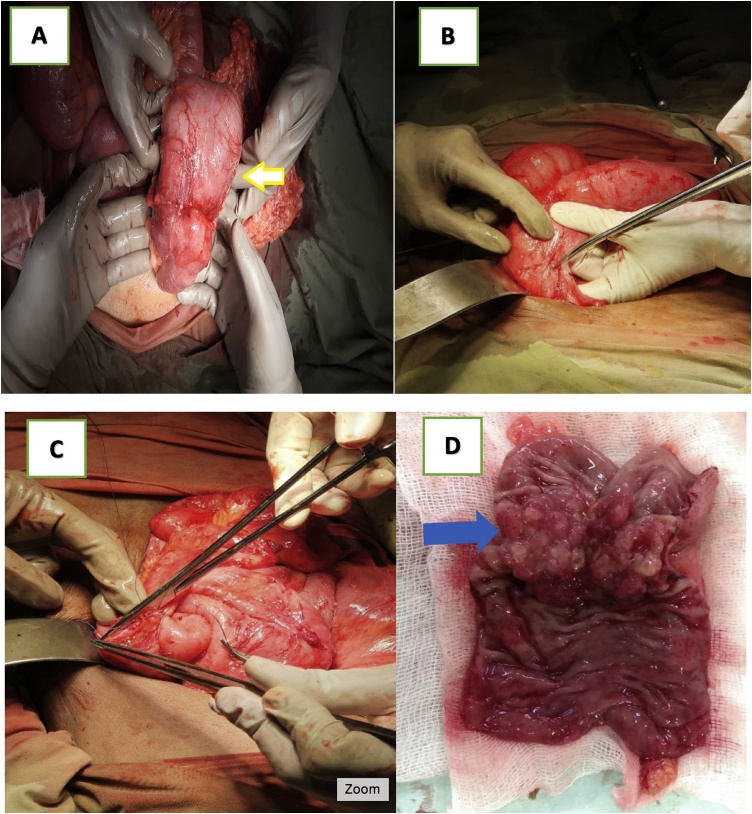
Intraoperative findings: A) showing the descending colon mass (yellow arrow); B) and C) left hemicolectomy with end-to-end anastomosis; D) gross photomicrograph of left hemicolectomy specimen showing a large ulcerated mass/growth around 7 × 6 × 5 cm infiltrating the serosa (blue arrow).

A 45-year-old woman came to the emergency room at our hospital with the chief complaints of abdominal distention and inability to defecate for seven days before entering the hospital on March 29th, 2018. Laboratory findings showed an elevated white blood cell count (12,480/μL), slightly decreased hemoglobin level (9.4 mg/dL), and CEA tumor markers 43.15 ng/m (high). A 3-position abdominal X-ray was performed and showed large bowel obstruction. Left hemicolectomy was performed with end-to-end anastomosis (see Fig. 2).

**Fig. 3:**
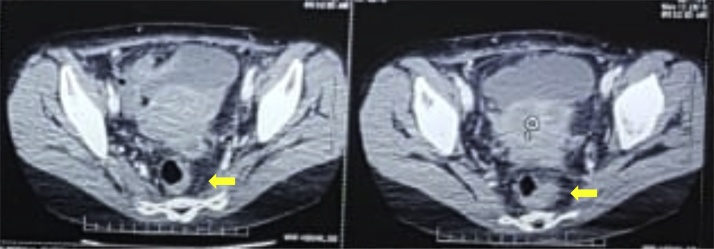
Preoperative MSCT scan with contrast shows large (7 cm) mass in descending colon tumor (yellow arrow).

**Fig. 4:**
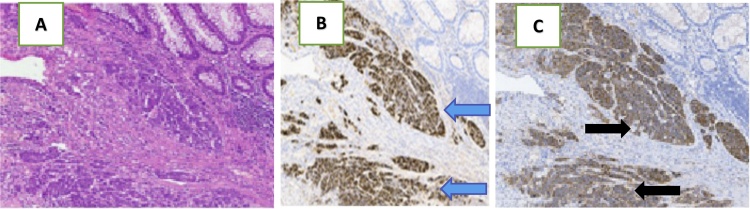
Histopathology slide showing: A) NEC infiltrating the submucosa (HE 4x); B) positive result for Chromogranin A in neoplastic cells (the nuclei are blue and the cytoplasm was brown) (blue arrows); C) positive result for the brown-stained cytoplasm for synaptophysin in neoplastic cells (black arrows).

**Fig. 5:**
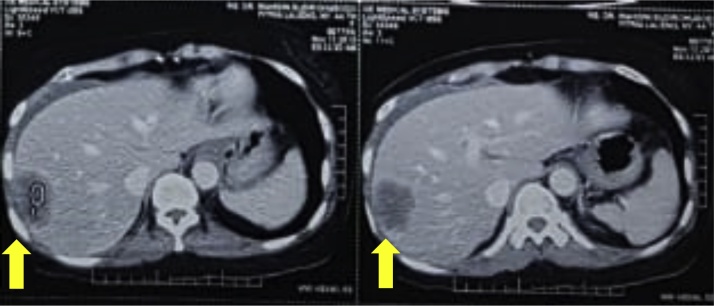
Surveillance MSCT scan with contrast shows hepatomegaly accompanied with right hepatic lobe lesion (yellow arrow).

Removal of a drain was performed on postoperative day 5, and the patient was discharged 8 days postoperative with a good condition. On March 23rd, 2018, prior to surgery, patient came to outpatient department in tertiary hospital with abdominal distention then an abdominal CT scan with contrast had been conducted that showed descending colon tumors (Fig. 3). The pathology report showed a malignant tumor, which showed neuroendocrine carcinoma (Fig. 4). From May 8th, 2018, until August 30th, 2018, the patient underwent five cycles of chemotherapy with 5 FU 700 mg, Cisplatin 98 mg, and octreotide acetate 20 mg, which was administered by the department of oncology in our institution. On November 8th, 2018, a chest x-ray showed normal limits and no sign of metastasis. On November 13th, 2018, a colon in loop showed a stenotic impression in the lower descending colon and colitis in the descending colon. Also on November 13th, 2018, plain abdominal x-ray was performed, with the impression that there were no radiological abnormalities in the abdomen. On November 15th, 2018, a whole abdominal MSCT was performed with the impression of minimal ascites and hepatomegaly accompanied with right hepatic lobe lesions (suggestive of metastatic tumors), as well circular thickening of descending colon (suggestive of descending colon tumors) (Fig. 5).

## 3 Discussion

With more than 100,000 cases, NET has a higher prevalence rate than gastric and pancreatic cancer [8]. The incidence of NET has increased by around 40–50 cases per million people per year. This may be due to improved diagnosis and the development of suitable, sensitive ways to measure this tumor, such as immunohistochemistry like chromogranin A (CgA) and diagnostic methods for tumor detection [6]. In the cases described—two GI-NET patients with symptoms of peritonitis and intestinal obstruction in Indonesia—accurate data regarding this tumor had not been obtained. The central hospital of Surabaya, Indonesia saw 5 cases over a period of 2 years (2008–2010) [9]. In contrast, our hospital saw 2 cases in 2018 alone. In most neuroendocrine tumors, the symptoms that appear are only a consequence of the growth of the tumor itself.

Data from the Surveillance, Epidemiology, and End Results (SEER) program indicated that the incidence of NET in the United States was 6.98 cases per 100,000 people in 2004 [6]. This analysis suggested that the rate of NET is increasing and that the prevalence of individuals with NET in the United States may exceed 170,000 [6]. Other independent studies in the SEER database also found that the incidence of GI NET increased during the period 1975–2008 [10]. The reason for this increase is uncertain, even though it appears that better diagnosis and classification is one factor [10]. Most NETs occur in the gastrointestinal tract (67.5%) and in the respiratory tract (25.3%). The most common locations of NETs in the GI tract are the small intestine (38%), rectum (34%), large intestine (16%), stomach (11%), and unknown sites (1%) [11].

NETs can be cancerous or benign; if NETs are malignant, the tumor has the potential to metastasize, although the tumor itself grows very slowly [5]. Symptoms tend to be vague and nonspecific, and these symptoms are often related to the mass effects of the tumor [4]. These can include vague abdominal colic, abdominal distention, weight loss, bleeding, obstruction, and constipation. However, the majority of the patients have no symptoms. Non-functional tumors can cause symptoms such as abdominal colic (68–78%), weight loss (32–50%), jaundice due to biliary obstruction or metastasis (21–50%), or vomiting (36%) [1,8].

When NET is present in the rectum, symptoms may include gastrointestinal bleeding, change in bowel habits, anorectal discomfort, and pruritus ani [12,13]. There may also be nonspecific local effects of the tumor. For example, fibrosis from carcinoid tumors may cause obstruction from adhesions or stricture of the intestinal lumen, hydronephrosis, and subsequent renal failure, or mesenteric ischemia from constriction of the mesenteric vessels [5]. The pathogenesis of this fibrosis is poorly recognized [14].

About 10% of patients with NETs will experience carcinoid syndrome, caused by the overproduction of serotonin or other hormones secreted by some NETs, ​​which often presents after the cancer spreads to other body parts [8]. Common symptoms are gut hypermotility (diarrhea), hot red flushing in the face, palpitations, and asthma attacks [14]. As stated, these symptoms occur in less than 10% of all patients, since the syndrome requires the presence of hepatic metastasis [15].

In our cases, the first patient, a 66-year-old man, presented with a history of sigmoidectomy and primary closure of a perforated caecum. The patient had a history of non-specific abdominal symptoms, including abdominal distention for three days and diarrhea for two weeks before being admitted to hospital. The tumor was found in the sigmoid colon at the time of the surgery and was confirmed by a histopathology study as a neuroendocrine tumor (Fig. 1). The history of this patient is consistent with the literature, in that some patients with NETs can experience abdominal distention and changes in bowel habits. In contrast to the first patient, our second patient, a 45-year-old woman, presented with abdominal distention and inability to defecate for seven days. This patient experienced obstructive symptoms of NET. Liver metastasis of descending colon tumors was also found approximately eight months after a left hemicolectomy.

Patients with suspected NETs should undergo biochemical evaluation. Traditionally, several plasma, serum, and urine markers have been evaluated as predictors of tumor progression in NETs, including 5-hydroxy indole acetic acid (5-HIAA), chromogranin A (CgA), serotonin, neuron-specific enolase (NSE), neurokinin A, E-cadherin, or neuropeptide K [16]. Currently, CgA and 5-HIAA are commonly used in the clinical routine for diagnosing and following up with patients with NETs [13,16]. The literature states that various tumor markers—including neuron-specific enolase (NSE) and chromogranin A (CgA)—have been evaluated as sources for detecting NETs and as indicators of tumor development and response to therapy [8]. Neuron-specific enolase has varying sensitivity (32%) and high specificity (100%) as a serum marker for NETs [17].

Elevated levels of CgA serum have been associated with poorer prognosis. CgA is sometimes used as a biochemical marker in non-functioning tumors. One meta-analysis calculated the sensitivity of CgA to range from 67% [17] to 73% [18] and its specificity to range from 86 [17] to 95% [18]. Increased circulating CgA levels are found in approximately 70% of NETs, both functional and non-functional—even in non-neoplastic cases, such as hepatic impairment, atrophic gastritis, and renal insufficiency. Gastritis patients with proton pump inhibitor (PPI) therapy can give false-positive results; therefore, in general, it should not be relied upon in isolation as a diagnostic test [19,20].

The most-commonly employed marker in patients suspected of having a carcinoid tumor is urinary 5-hydroxy indole acetic acid (5-HIAA). Serotonin is a neurotransmitter that is produced mainly in the brain but also in the bronchi and gastrointestinal tract; it is degraded in the liver, and its metabolite 5-HIAA is excreted through urine [20]. Usually, 5-HIAA is only present in small amounts in the urine, but it can increase in carcinoid syndrome. The 5-HIAA sensitivity was seen to be 65–75% with a specificity of 100% [17].

Imaging has a vital role in locating the primary tumor, identifying the location of metastasis site(s), and assessing the response to treatment [9]. In this case series, the initial imaging was used to determine tumor reaction and to evaluate the resectability of the tumor. Imaging included an ultrasound examination, computed tomography (CT), and magnetic resonance imaging (MRI) [13]. The European Neuroendocrine Tumor Society (ENETS) guidelines recommend using CT or MRI for patients with tumors larger than 10 mm in size and for recurrence and suspected metastasis to other organs [13].

According to the World Health Organization (WHO)’s classification (2019), NET is determined by histopathology and biological characteristics, including grading of tumor cells, size and location of primary tumors, a proliferation of tumor cell markers, local and vascular invasion, and production of active biological substances (Table 1). The classification includes well-differentiated endocrine tumors (low grade = G1, intermediate grade = G2, high grade = G3) and poorly-differentiated endocrine tumors (high grade = G3). Tumor differentiation and tumor grade often correlate with mitotic count and Ki-67 proliferation index [21]. The most commonly used histologic classification schemes include both the European Neuroendocrine Tumor Society and WHO systems, incorporate mitotic rate and Ki-67 index [22].

**Table 1:** WHO Classification of NET (2019) [20].

Differentiation	Grade	GI-NET (excluding pancreas)
Well-differentiated	Low Grade (G1)	<2 mitoses/10 HPF and/or <3% Ki-67 index
	Intermediate Grade (G2)	2–20 mitoses/10 HPF and/or 3–20% Ki-67 index
	High Grade (G3)	>20 mitoses/10 HPF and/or >20% Ki-67 index
Poorly differentiated	High Grade (G3)	>20 mitoses/10 HPF and/or >20% Ki-67 index

The characteristics of the NETs in our patients were described in the histopathology report, which is summarized in Table 2. Histopathology study of both patients did not include the Ki-67 index in the examination.

**Table 2:** The Histopathology and Biological Characteristics of the NETs in Our Cases.

Histopathology examination	Case 1	Case 2
Anatomical site of tumor	Sigmoid colon	Descending colon
Diagnosis	Peritonitis caused by caecum perforation	Mechanical bowel obstruction
Grade	cT4N0M0 (stage IIIA)	cT4N0M1 (stage IV)
Mitotic rate	High mitotic rate	Low mitotic rate
Size of tumor	4 × 10 cm	7 × 6 × 5 cm
Presence of multiform disease	Negative	Negative
Presence of vascular invasion	Negative	Negative
Presence of perineural invasion	Negative	Negative
Lymph node metastasis	Negative	Negative
Margin status	Positive	Positive

The American Joint Committee on Cancer (AJCC) Manual, 8th edition, has created staging definitions according to T, N, M [23]. The definitions and the TNM Staging System for Neuroendocrine Tumors in the Colon and Rectum (carcinoid tumors) are found in Table 3, Table 4.

**Table 3:** Definitions for T, N, M.

T	PRIMARY TUMOR
Tx	Primary tumor cannot be assessed
T0	No evidence of primary tumor
T1	Tumor invades the lamina propria or submucosa and is ≤2 cm
T1a	Tumor <1 cm in greatest dimension
T1b	Tumor 1–2 cm in greatest dimension
T2	Tumor invades the muscularis propria or is >2 cm with invasion of the lamina propria or submucosa.
T3	Tumor invades through the muscularis propria into subserosa tissue without penetration of overlying serosa.
T4	Tumor invades the visceral peritoneum (serosa) or other organs or adjacent structures.

	*Note: For any T, add “(m)” for multiple tumors [TX (#) or TX(m), where X = 1−4 and # = number of primary tumors identified**]; for multiple tumors with different T, use the highest. **Example: If there are two primary tumors, only one of which invades through the muscularis propria into the subserosa tissue without penetration of the overlying serosa, we define the primary tumor as either T3(2) or T3(m).

N	REGIONAL LYMPH NODES

Nx	Regional lymph nodes cannot be assessed
N0	No regional lymph node metastasis
N1	Regional lymph node metastasis

M	DISTANT METASTASIS

M0	No distant metastasis
M1	Distant metastasis
M1a	Metastasis confined to liver
M1b	Metastases in at least one extrahepatic site (e.g., lung, ovary, nonregional lymph node, peritoneum, bone)
M1c	Both hepatic and extrahepatic metastases

**Table 4:** AJCC Prognostic Group.

	T	N	M
Stage I	T1	N0	M0
Stage IIA	T2	N0	M0
Stage IIB	T3	N0	M0
Stage IIIA	T4	N0	M0
Stage IIIB	T1	N1	M0
	T2	N1	M0
	T3	N1	M0
	T4	N1	M0
Stage IV	Tx, T0	Any N	M1
	T1	Any N	M1
	T2	Any N	M1
	T3	Any N	M1
	T4	Any N	M1

We classified the first patient as cT4N0M0 (stage IIIA) based on clinical characteristics and as pT4N0M0 (stage IIIA) according to the histopathology findings. Unlike the first patient, the second patient had a history of liver metastasis with ascites, gall bladder stones, and hepatomegaly, which was found in surveillance eight months after the initial surgery. We classified this patient as cT4N0M1 (stage IV).

If possible, radical surgery is the standard therapy for NETs. If there is locoregional or liver metastasis, debulking surgery can be performed, with the possibility of the tumor being removed at almost 90% [24]. It is recommended to perform palliative surgery in the following clinical situations: in primary tumors with non-operable liver metastases (especially functional NETs), because symptoms correlate with neoplastic masses; if the primary tumor is localized in the small intestine, because it can cause intestinal obstruction; and in the context of surgery that allows for subsequent multimodal treatment [25].

Resection of the GI-NET must be followed by adequate regional lymph node resection (including all palpable tumors, where feasible) and thorough exploration of the associated synchronous primary tumors (15%–30% incidence) [25].

In the first case, we performed Hartmann’s procedure and primary closure on the perforated caecum, because the tumor was located in the sigmoid colon. For our second patient, we performed left hemicolectomy with end-to-end anastomosis, because of the descending colon tumor. This surgical treatment was consistent with NCCN recommendations for locoregional disease—that is, bowel resection with regional lymphadenectomy.

Within 3–12 months after the operation, it is necessary to perform GI-NET surveillance, which consists of history-taking and physical examination, biochemical marker examination (as clinically indicated), abdominal pelvic multiphasic CT or MRI (as clinically indicated), and chest CT with or without contrast (as clinically indicated).

Both of our patients underwent surveillance within one year after the resection. The first patient underwent surveillance at three months after the resection. The surveillance included abdominal ultrasound (which showed no signs of metastasis), chest x-ray (which found no sign of metastasis), and lopography results (which were within normal limits with a distance of 9.5 cm between the proximal and distal colon). Our second patient underwent surveillance at eight months after the resection. The surveillance included chest x-ray (which showed the heart and lungs were within normal limits), colon in loop (which showed colitis and stenosis in the lower descending colon), and whole abdominal MSCT (which showed minimal ascites, gallbladder stones, and right hepatic lesions, which is suggestive of metastatic tumor).

The prognosis for patients with NETs varies according to the stage at diagnosis, the histologic classification, and the primary site of the tumor. Tumors measuring > 2 cm with invasion of muscularis propria at the time of diagnosis has an overall poor prognosis, with 5-year survival rates of only 33–42%. The first patient was classified as stage IIIA with the following histopathology characteristics: all lymph nodes were free of tumors, high mitotic rate, and the primary site of the tumor was in the sigmoid colon. The second patient was classified as stage IV with liver metastasis, the histopathology characteristic of low mitotic rate, and the primary site being in descending colon. Both of the patients had a poor prognosis due to muscularis propria invasion and thus had the 5-year survival rates mentioned above.

## 4 Conclusion

Two uncommon cases of colon and sigmoid colon carcinoma with neuroendocrine features in patients with peritonitis and large bowel obstruction were described. Because of the rapid growth pattern of GI-NET, it is difficult to manage; therefore, early diagnosis, careful management, and thorough understanding of the disease are very important. Staging and classification systems for GI-NETs are likely to continue to evolve, along with further advancement of tumor-directed diagnostic and therapeutic modalities, as our understanding of GI-NET continues to grow over time. We hope that our case series can enhance the information related to GI-NETs and help clinicians better understand this disease.

## Declaration of Competing Interest

Nothing to declare.

## Funding

No funding or sponsorship.

## Ethical approval

The study is exempt from ethical approval in our institution.

## Consent

Written informed consent was obtained from the patient for publication of this case report and accompanying images. A copy of the written consent is available for review by the Editor-in-Chief of this journal on request.

## Registration of research studies

Not applicable – case series report.

## Guarantor

Warsinggih.

## Provenance and peer review

Not commissioned, externally peer-reviewed.

## CRediT authorship contribution statement

**Warsinggih:** Conceptualization, Methodology, Supervision. **Liliyanto:** Conceptualization, Methodology, Data curation, Writing - original draft, Writing - review & editing. **Prihantono:** Data curation, Writing - original draft, Supervision. **Gusti Deasy Wilda Ariani:** Visualization, Investigation. **Muhammad Faruk:** Software, Validation, Writing - review & editing.

## References

[1] Aytac E., Ozdemir Y., Ozuner G. (2014). Long term outcomes of neuroendocrine carcinomas (high-grade neuroendocrine tumors) of the colon, rectum, and anal canal. J. Visc. Surg..

[2] Yoshida T., Kamimura K., Hosaka K., Doumori K., Oka H., Sato A., Fukuhara Y., Watanabe S., Sato T., Yoshikawa A., Tomidokoro T., Terai S. (2019). Colorectal neuroendocrine carcinoma: a case report and review of the literature. World J. Clin. Cases.

[3] André T.R., Brito M., Freire J.G., Moreira A. (2018). Rectal and anal canal neuroendocrine tumours. J. Gastrointest. Oncol..

[4] Mussan-Chelminsky G., Vidal-González P., Núñez-García E., Valencia-García L.C., Márquez-Ugalde M.Á. (2015). Intestinal carcinoid tumour: case report. Cirugía Cir..

[5] Alshammari T.F., Hakami R.A., Alali M.N., AlShammari S., Zayed M.A., AlSohaibani M.O., Bin Traiki T. (2019). A perforated colonic neuroendocrine tumor with liver metastasis: a case report and literature review. Am. J. Case Rep..

[6] Dasari A., Shen C., Halperin D., Zhao B., Zhou S., Xu Y., Shih T., Yao J.C. (2017). Trends in the incidence, prevalence, and survival outcomes in patients with neuroendocrine tumors in the United States. JAMA Oncol..

[7] Agha R.A., Borrelli M.R., Farwana R., Koshy K., Fowler A.J., Orgill D.P., Zhu H., Alsawadi A., Noureldin A., Rao A., Enam A., Thoma A., Bashashati M., Vasudevan B., Beamish A., Challacombe B., De Wilde R.L., Machado-Aranda D., Laskin D., Muzumdar D., D’cruz A., Manning T., Healy D., Pagano D., Goel P., Ranganathan P., Pai P.S., Raja S., Ather M.H., kadioäžlu H., Nixon I., Mukherjee I., Gómez Rivas J., Raveendran K., Derbyshire L., Valmasoni M., Chalkoo M., Raison N., Muensterer O., Bradley P., Roberto C., Afifi R., Rosin D., Klappenbach R., Wynn R., Giordano S., Basu S., Surani S., Suman P., Thorat M., Kasi V. (2018). The PROCESS 2018 statement: Updating Consensus Preferred Reporting Of CasE Series in Surgery (PROCESS) guidelines. Int. J. Surg..

[8] Carlini M., Appetecchia M., Carlini M. (2018). Neuroendocrine tumors: a nosologic framework. Abdom. Neuroendocr. Tumors.

[9] Mahayasa M., Wibowo P.S. (2018). Tumor Neuroendokrin: Kasus Serial di RSUD Dr. Soetomo. JBN (Jurnal Bedah Nasional)..

[10] Tsikitis V.L., Wertheim B.C., Guerrero M.A. (2012). Trends of incidence and survival of gastrointestinal neuroendocrine tumors in the United States: a seer analysis. J. Cancer.

[11] Yao J.C., Hassan M., Phan A., Dagohoy C., Leary C., Mares J.E., Abdalla E.K., Fleming J.B., Vauthey J.-N., Rashid A., Evans D.B. (2008). One hundred years after “carcinoid”: epidemiology of and prognostic factors for neuroendocrine tumors in 35,825 cases in the United States. J. Clin. Oncol..

[12] Estrozi B., Bacchi C.E. (2011). Neuroendocrine tumors involving the gastroenteropancreatic tract: a clinicopathological evaluation of 773 cases. Clinics (Sao Paulo).

[13] Rodrigues Â., Castro-Poças F., Pedroto I. (2015). Neuroendocrine rectal tumors: main features and management, GE port. J. Gastroenterol..

[14] Laskaratos F.-M., Rombouts K., Caplin M., Toumpanakis C., Thirlwell C., Mandair D. (2017). Neuroendocrine tumors and fibrosis: an unsolved mystery?. Cancer.

[15] Druce M., Rockall A., Grossman A.B. (2009). Fibrosis and carcinoid syndrome: from causation to future therapy. Nat. Rev. Endocrinol..

[16] Koenig A., Krug S., Mueller D., Barth P.J., Koenig U., Scharf M., Ellenrieder V., Michl P., Moll R., Homayunfar K., Kann P.H., Stroebel P., Gress T.M., Rinke A. (2017). Clinicopathological hallmarks and biomarkers of colorectal neuroendocrine neoplasms. PLoS One.

[17] Bajetta E., Ferrari L., Martinetti A., Celio L., Procopio G., Artale S., Zilembo N., Di Bartolomeo M., Seregni E., Bombardieri E. (1999). Chromogranin A, neuron specific enolase, carcinoembryonic antigen, and hydroxyindole acetic acid evaluation in patients with neuroendocrine tumors. Cancer.

[18] Al-Risi E.S., Al-Essry F.S., Mula-Abed W.-A.S. (2017). Chromogranin a as a biochemical marker for neuroendocrine tumors: a single center experience at Royal Hospital, Oman. Oman Med. J..

[19] Campana D., Nori F., Piscitelli L., Morselli-Labate A.M., Pezzilli R., Corinaldesi R., Tomassetti P. (2007). Chromogranin A: is it a useful marker of neuroendocrine tumors?. J. Clin. Oncol..

[20] Massironi S., Sciola V., Peracchi M., Ciafardini C., Spampatti M.P., Conte D. (2008). Neuroendocrine tumors of the gastro-entero-pancreatic system. World J. Gastroenterol..

[21] Nagtegaal I., Odze R., Klimstra D., Paradis V., Rugge M., Schirmacher P., Washington M., Carneiro F., Cree I. (2019). The 2019 WHO classification of tumours of the digestive system. Histopathology.

[22] Cavalcanti M.S., Gönen M., Klimstra D.S. (2016). The ENETS/WHO grading system for neuroendocrine neoplasms of the gastroenteropancreatic system: a review of the current state, limitations and proposals for modifications. Int. J. Endocr. Oncol..

[23] Amin M.B., Greene F.L., Edge S.B., Compton C.C., Gershenwald J.E., Brookland R.K., Meyer L., Gress D.M., Byrd D.R., Winchester D.P. (2017). The Eighth Edition AJCC Cancer Staging Manual: Continuing to build a bridge from a population-based to a more “personalized” approach to cancer staging. CA Cancer J. Clin..

[24] Bajetta E., Catena L., Procopio G., Bichisao E., Ferrari L., Della Torre S., De Dosso S., Iacobelli S., Buzzoni R., Mariani L., Rosai J. (2005). Is the new WHO classification of neuroendocrine tumours useful for selecting an appropriate treatment?. Ann. Oncol. Off. J. Eur. Soc. Med. Oncol..

[25] Shah M.H., Goldner W.S., Halfdanarson T.R., Bergsland E., Berlin J.D., Halperin D., Chan J., Kulke M.H., Benson A.B., Blaszkowsky L.S., Eads J., Engstrom P.F., Fanta P., Giordano T., He J., Heslin M.J., Kalemkerian G.P., Kandeel F., Khan S.A., Kidwai W.Z., Kunz P.L., Kuvshinoff B.W., Lieu C., Pillarisetty V.G., Saltz L., Sosa J.A., Strosberg J.R., Sussman C.A., Trikalinos N.A., Uboha N.A., Whisenant J., Wong T., Yao J.C., Burns J.L., Ogba N., Zuccarino-Catania G. (2018). NCCN guidelines insights: neuroendocrine and adrenal tumors, version 2.2018. J. Natl. Compr. Canc. Netw..

